# Patient-Centered Care: Transforming the Health Care System in Vietnam With Support of Digital Health Technology

**DOI:** 10.2196/24601

**Published:** 2021-06-04

**Authors:** Thu Ha Dang, Tuan Anh Nguyen, Minh Hoang Van, Olinda Santin, Oanh Mai Thi Tran, Penelope Schofield

**Affiliations:** 1 Department of Psychological Sciences School of Health Sciences Swinburne University of Technology Melbourne Australia; 2 Behavioural Sciences Unit Health Services Research and Implementation Sciences Peter MacCallum Cancer Centre Melbourne Australia; 3 Digital Health Cooperative Research Centre Sydney Australia; 4 Social Gerontology Division National Ageing Research Institute Melbourne Australia; 5 Quality Use of Medicines & Pharmacy Research Centre Clinical and Health Sciences University of South Australia Adelaide Australia; 6 Health Strategy and Policy Institute Hanoi Vietnam; 7 Hanoi University of Public Health Hanoi Vietnam; 8 National Institute of Health Sciences Bach Mai Hospital Hanoi Vietnam; 9 School of Nursing and Midwifery Medical Biology Centre Queen's University Belfast Belfast United Kingdom; 10 Department of Psychology, and Iverson Health Innovation Research Institute Swinburne University of Technology Melbourne Australia; 11 Sir Peter MacCallum Department of Oncology The University of Melbourne Melbourne Australia

**Keywords:** building blocks, digital health, eHealth, patient-centered care, telemedicine, Vietnam

## Abstract

**Background:**

Over the recent decades, Vietnam has attained remarkable achievements in all areas of health care. However, shortcomings including health disparities persist particularly with a rapidly aging population. This has resulted in a shift in the disease burden from communicable to noncommunicable diseases such as dementia, cancer, and diabetes. These medical conditions require long-term care, which causes an accelerating crisis for the health sector and society. The current health care system in Vietnam is unlikely to cope with these challenges.

**Objective:**

The aim of this paper was to explore the opportunities, challenges, and necessary conditions for Vietnam in transforming toward a patient-centered care model to produce better health for people and reduce health care costs.

**Methods:**

We examine the applicability of a personalized and integrated Bespoke Health Care System (BHS) for Vietnam using a strength, weakness, opportunity, and threat analysis and examining the successes or failures of digital health care innovations in Vietnam. We then make suggestions for successful adoption of the BHS model in Vietnam.

**Results:**

The BHS model of patient-centered care empowers patients to become active participants in their own health care. Vietnam’s current policy, social, technological, and economic environment favors the transition of its health care system toward the BHS model. Nevertheless, the country is in an early stage of health care digitalization. The legal and regulatory system to protect patient privacy and information security is still lacking. The readiness to implement electronic medical records, a core element of the BHS, varies across health providers and clinical practices. The scarcity of empirical evidence and evaluation regarding the effectiveness and sustainability of digital health initiatives is an obstacle to the Vietnamese government in policymaking, development, and implementation of health care digitalization.

**Conclusions:**

Implementing a personalized and integrated health care system may help Vietnam to address health care needs, reduce pressure on the health care system and society, improve health care delivery, and promote health equity. However, in order to adopt the patient-centered care system and digitalized health care, a whole-system approach in transformation and operation with a co-design in the whole span of a digital health initiative developing process are necessary.

## Introduction

### Overview

Following broad economic reforms known as Doi Moi in 1986, Vietnam has attained remarkable health care improvement, reflected in core health indicators [1]. From 1988 to 2018, life expectancy at birth increased from 69.9 years to 75.3 years, under-5 mortality rate decreased from 56‰ to 20.7‰, and infant mortality reduced from 39.6‰ to 16.5‰ [2]. Health care expenditure gradually increased and was forecasted to triple from US $15.6 billion in 2018 to US $42.9 billion in 2028 [3]. Despite these improvements, the health care system still faces significant challenges including wide disparities in health and growing health care costs. The disparities in core health indicators are particularly observed between urban and rural residents, across different regions, and among population groups [4]. For example, the maternal mortality ratio and infant mortality rate in some mountainous areas are 3 to 4 times higher than those in lowland and urban areas and almost double the national average rates [4].

Vietnam is undergoing a dramatic demographic transition resulting in an aging population. The number of people aged 65 years and over is estimated to increase from 10% of the population in 2015 to 28% in 2050 [5]. The combination of an aging population, increased industrialization, and changes in population lifestyle have created a double disease burden, with a shift from communicable to noncommunicable diseases (NCDs). Specifically, the mortality rate caused by NCDs rose from 45.5% in 2010 to 77% in 2016 and is projected to climb [3,4,6]. The double disease burden means that Vietnam is facing more costly health conditions such as dementia, cancer, and multimorbidity. In addition to this, the country still faces significant burden of infectious conditions and a number of new epidemics such as COVID-19. These health challenges must be addressed using a systemic approach by the whole government of Vietnam to improve health for its population.

**Figure 1:**
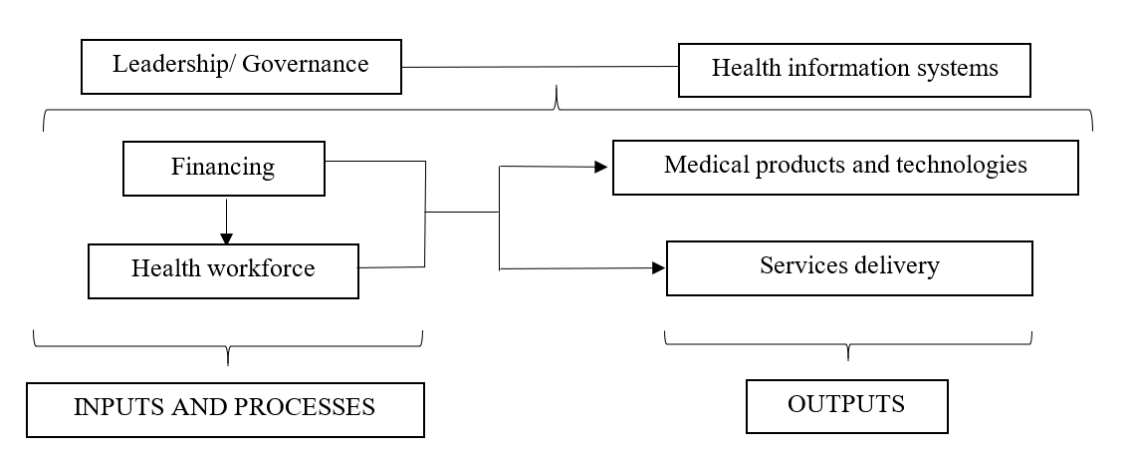
Health system strengthening: relationship between the World Health Organization building blocks (6 core components, top) and International Health Partnership monitoring and evaluation framework (inputs, processes and outputs, bottom).

To improve health care problems and the health status of the population, there is a critical need for a well-functioning health care system that can deliver services equitably and efficiently [7]. The World Health Organization (WHO) developed an evidence-based building blocks framework as a tool to help its member states analyze their health care systems. This framework allows nations to consider the multifaceted nature of their health systems and interdisciplinary and multilevel responsibility in health care [7]. The WHO framework assesses health systems using 6 core components or building blocks. Each building block and its indicators were initiated by a group of agency representatives and technical experts, shared broadly with country experts, and followed by evaluations through a series of case studies and reviews of country experiences [7]. These components focus on the key chains of the monitoring and evaluation framework developed by the International Health Partnership, namely inputs, processes, and outputs [8]. The relation between the 6 building blocks and this monitoring and evaluation framework is summarized in Figure 1. In the following section, the 6 building blocks and corresponding indicators are used to describe Vietnam’s health care system, comprising financing and health workforce components (inputs and processes), medical products, technologies, and service delivery components (immediate outputs), and cross-cutting components: leadership/governance and health information systems [7].

### Health Care System in Vietnam

#### Health Financing

Health financing is a key building block in a national health system, largely influencing the inputs, thus affecting the availability, affordability, and accessibility of health services. A good health financing system should move toward universal health coverage, where all people have access to needed health services without financial hardship. This could be achieved through increasing total health expenditure (THE) and decreasing the proportion of households facing financial catastrophe as a result of out-of-pocket payments (OPP) [7].

With multiple health financing reforms, Vietnam’s THE per capita increased from US $14 in 1995 to US $113 in 2014 [9,10], thus within the internationally defined range and enough for universal coverage of key health interventions [7]. The increase in public health expenditure (mainly comprising state budget [11] and social health insurance [12]) has increased health care coverage for some groups including the poor, ethnic minorities, under–6-year-old children, over–80-year-old people, and socially vulnerable groups through the government’s subsidized schemes [13]. However, patient OPP remained high, accounting for 40.8% of THE in 2015, which was higher than that of other countries in the Asia Pacific region and the WHO recommended level [10,11]. The high OPP led to catastrophic expenditure and pushed many Vietnamese families into poverty, resulting in health care inequity [14,15]. The current model of the health care system and financing needs further reforms to address a surge in health care expenditure caused by the aging population and shifting disease pattern in Vietnam.

#### Health Workforce

The health workforce is another key building block to provide inputs and processes to the monitoring and evaluation chain of health systems. The ability of a country to meet its health goals largely depends on people in charge of organizing and delivering health services. Evidence of the direct and positive link between numbers of health workers and population health outcomes has been demonstrated in several studies [16,17].

The health workforce in Vietnam has gradually improved in both quantity and quality. The number of doctors and pharmacists increased from 7.2 and 1.76 per 10,000 people in 2010 to 8.0 and 2.2 per 10,000 people in 2015, respectively [4]. In 2015, 65% to 95% of the health facilities and about 90% of the health workers in hospitals at central and provincial levels were licensed [4]. Despite this significant improvement, Vietnam’s health workforce was still insufficient to meet staffing norms and clinical needs [4] and inappropriately distributed across regions and levels/areas of care. The aging population and shifting disease burden to those requiring long-term care for chronic NCDs are likely to lead to severe shortages in health resources, which occur in highly specialized fields such as cancer, palliative care, and mental health and in hard-to-reach areas such as North West, Central highlands, and Mekong Delta regions [4,18-20]. The mountainous and remote areas lack not only specialists trained in advanced diagnostic and treatment approaches but also standard medical and diagnostic equipment, which diminishes the quality of health care services in these areas compared to urban regions [4]. This wide disparity in health care between the rich and the poor, urban and rural, is demonstrated in the disparity of core health indicators such as life expectancy at birth and infant and under-5 mortality rates [4].

Overcrowding in health facilities, especially in urban and specialized hospitals, is a main cause of health worker exhaustion. Nearly one-fifth of Vietnamese clinical nurses experienced burnout and occupational stress [21]. Clinician burnout directly reduces the quality of life of clinicians and adversely affects the quality of care to patients. It also indirectly contributes to the reduction of health staffing [22]. Hence, a vicious negative cycle for Vietnam’s health sector is created.

#### Health Service Delivery

Health service delivery is reflected in the availability and readiness of services across the health care continuum. Vietnam has achieved significant service improvement in health care: for example, the ratio of hospital beds per 10,000 people increased from 21.5 in 2011 to 24.0 in 2015 [4]. The hospital quality management system was established in 2013 and available in 55.4% of hospitals throughout the country in 2015 [23].

Service provision is immediate outputs of the inputs into the health system such as financing, workforce, procurement, and supplies [7]. It will be difficult to achieve the outputs if the inputs are insufficient. Even if the inputs are adequate, whether the outputs are obtained depends very much on the efficiency of the health system’s functioning. According to Bentley et al [24], there are 4 key inefficiencies: duplication of services, inefficient processes, overly expensive inputs, and medical errors. All of these forms of inefficiency occur in Vietnam’s health sector.

Vietnamese patients’ laboratory tests and results are not usually archived at medical facilities or shared between different health care providers. This poses a challenge as patients experience multiple visits to doctors and specialists for the same health conditions thus leading to duplication of services [3]. Public hospitals, especially in large cities, are usually overcrowded with 2 to 3 patients sharing a bed. Limited quality of health care services at the commune level leads to reduced patient trust in primary care. Also, if patients use OPP, the health care system allows them to easily bypass lower level facilities (eg, commune health stations) and seek health care services in leading tertiary hospitals in big cities without referrals (ie, inefficient processes), even just to treat common diseases that primary care is well equipped to manage (ie, overly expensive inputs) [4,25]. Consequently, higher level hospitals are drained of resources, while there is waste at lower levels due to underuse [26]. Overcrowding in high-level health facilities is associated with medication errors. A large prospective study in two urban hospitals in Vietnam revealed that medication errors occurred in more than one-third of all medication doses [27].

There are also discrepancies in health service readiness and quality across areas of health care. Vietnam has an extensive primary health care system that reaches almost every administrative jurisdiction and acts as the main entry point to public health care. However, grassroots level facilities have inadequate infrastructure required for basic health care delivery. For example, only 76% of commune health stations in Dien Bien province (a Northern mountainous province) have a source of clean water, and a significant number of district hospitals lack essential equipment such as child ventilators and electrocardiograms [28]. Also, there have been increasing concerns about the equity and quality of basic health service provisions in primary health care. These limitations were reflected in key health indicators, with the infant mortality rate among the ethnic minority population being over 4 times higher than that of Kinh and Hoa ethnic groups, who mostly live in urban areas [28].

#### Access to Essential Medicines

According to WHO, a well-functioning health system should be able to provide the population it serves with equitable and affordable access to essential medicines, medical products, and technologies and use this resource efficiently [7]. However, published literature consistently reported that medicine prices in Vietnam were high and unaffordable for many Vietnamese people [29-31]. A fragmented medical information system within and between health care and other sectors such as General Department of Vietnam Customs and Ministry of Finance, as well as a shortage of personnel and resources for enforcing medicine pricing policies was one of the reasons for their high price in Vietnam [32]. Because of high medicine prices, irrational selection and use of medicines, unsustainable pharmaceutical production and distribution systems, and a lack of financial support systems for medicine procurement, access to the right medicines at the times people need them remains a major challenge for the majority of the Vietnamese people [33-37].

#### Health Information

The health information system (HIS) is a crosscutting building block because it creates a foundation for all decision-making processes in a health system. Vietnam has achieved significant progress in this area. A number of health statistics are generated annually: for example, the Annual Health Statistic Yearbook, Joint Annual Health Review, and Statistical Yearbook. Information technology is applied widely in the health administration and management of all 63 provinces and cities across the country. Vietnam is also strongly promoting the development of a health management database for its over 90 million citizens [4].

Vietnam’s HIS, however, still faces a number of issues and challenges, including data generation, validation, and uses. For example, data from health care facilities, especially from private and industrial sectors, are neither timely nor complete. Information from local death registration is incompatible with WHO recommendations. There are discrepancies in the quality of medical records across regions and levels of care, which creates challenges for continuing health monitoring, disease prevention and treatment, and management of medical errors and adverse drug reactions. Even after the data are collected, the unclear data dissemination mechanism in Vietnam will likely restrict data use [4]. Given the importance of this crosscutting building block of the health system, reform is needed to operate HIS more effectively, especially in the new era of information technology.

#### Leadership and Governance

Health leadership and governance is another crosscutting building block in a health care system. It mediates other building blocks by connecting all issues surrounding the accountability of various stakeholders in the system to ensure adequate resources (finance, workforce, medical supplies, and information) are available to deliver essential health services. In terms of health care governance, Vietnam has attained remarkable progress, demonstrated in the development of a strong policy framework in health care. The enactment of important laws and policies, such as health insurance and pharmacy laws [38,39], and the Health Sector Strategy for 2011-2020 with a Vision to 2030 [40] provided a solid foundation and led to the formation of regulations, guidelines, initiatives, and plans in these areas. The organizational structure of Vietnam’s health system has been adjusting to meet health care needs at various levels, such as establishing information technology administration at central levels and formally affirming the function and tasks of community health services at local levels [4]. Nevertheless, health policies in Vietnam are sometimes overlap, inconsistent and lack evidence. Lack of detailed plans and information cause difficulty for the effective implementation of policies. Additionally, insufficient sanctions and a weak inspection network for policy enforcement are also shortcomings of Vietnam’s health care governance [4].

In summary, Vietnam has attained significant achievement and improvement in all 6 areas of the health system framework. However, shortcomings persist. To address all these systemic challenges and achieve optimal and equitable health outcomes for the population, the Vietnamese government must consider adapting the current model of health care operation to meet rapidly changing population trends, patterns of disease, and health care needs and use existing resources more efficiently while improving health infrastructure. The application of digital health technologies to underpin the health care system transformation is critical to success [41]. “The use and scale-up of digital health solutions can revolutionize how people worldwide achieve higher standards of health and access services to promote and protect their health and well-being” [42]. As in many other countries, Vietnam’s health care system will need to be migrated to the patient-centered care model, which focuses on patients and their particular health care needs, and patients will need to be empowered to become active participants in their care to optimize health and economic outcomes [43]. This study was conducted to explore the opportunities, challenges, and necessary conditions for Vietnam in transforming toward a patient-centered care model to produce better health for people and reduce health care costs.

## Methods

### Approach

In this paper, we explored a Bespoke Health Care System (BHS) developed by Schofield et al [44] as an ideal example of a comprehensive patient-centric care model. The current Vietnamese digital health and health care landscape was examined using a strength, weakness, opportunity, and threat (SWOT) analysis to argue for the potential of developing a BHS in Vietnam. We then discuss necessary and sufficient conditions and challenges for Vietnam in transforming toward this patient-centered care model to produce better health for people and reduce health care costs.

### Bespoke Health Care System

In 2019, Schofield et al [44] first introduced the concept of the BHS in the context of Australian health care, which has been adapted to other contexts [45,46]. The development of the BHS was based on the pedagogical model of flipping the classroom in modern education [47] and its application in health care [48]. In Australia, this approach has been applied by many health professionals [47]. Patients were equipped with necessary knowledge before consultations. Therefore, the consultation time was effectively used to solve health problems and make joint decisions [47]. The core component of the BHS is “increasing patient involvement in health care decisions and self-management assisted by the use of technology” [49]. In this model, patients will be educated about their illness and management options, flipping their role from passengers to drivers to manage their own health care, with clinicians playing more of a support role being the “guide by the side.” Although the BHS was proposed as an ideal comprehensive patient-centric care system with several advantages and benefits, there is no one-size-fits-all approach. Novel health care solutions will work best when they are adapted to suit a country’s specific conditions and have broad-based acceptance among the community, health care providers, and government agencies.

### SWOT Analysis

The SWOT analysis has been used widely in policy research to provide policy makers with a sound basis for strategy development and formulation and identify new avenues for national health care reform [50]. The technique examines 4 parameters: strengths, weaknesses, opportunities, and threats. In this paper, strengths and weaknesses refer to internal factors of the proposed BHS that place it at an advantage or disadvantage over the current Vietnamese health care system, respectively. Opportunities and threats refer to external factors to the BHS that support or prevent it from being adopted in Vietnam.

## Results

### Strengths

The BHS brings a number of potential benefits to a health care system. First, the BHS proposes integrating electronic medical records (EMRs) into a patient-centered management platform. Currently, EMRs act as passive information depositories mainly for the purposes of health data storage or analysis. In the proposed BHS, every person would have their EMR containing all relevant personal and medical information that is shared across health care providers. As such, relevant health workers and agencies would have access to patients’ real-time health information, saving time in managing people’s health, saving costs of unnecessary or duplicated examinations and laboratory tests, and reducing medication errors. Studies have revealed that one-fifth of medical errors were due to insufficient patient medication information [48,51]. Clinical access to patients’ real-time health information would enable tailored precision and holistic health solutions for individuals.

Second, the BHS proposes that the EMR is an active tool in promoting optimal health management. The proposed platform would remotely track symptoms, prompt patients to perform routine screening tests, allow patients to book medical appointments and remind them to attend, and provide scheduled reminders for adherence to medical recommendations. Electronic prompts and reminder alerts have been shown to assist individuals in adhering to clinical intervention effectively, particularly in managing long-term chronic conditions [49].

Third, the BHS serves as an education platform, upskilling patients and health workers. In the BHS, patients would optimally be equipped with a comprehensive understanding of their conditions, treatment options, and self-management strategies. This is an effective way to empower people to have greater ownership in managing their health. Additionally, clinicians would be provided with up-to-date, evidence-based optimal health care pathways suitable for their specific patients’ conditions. This offers an ongoing mechanism to support clinicians’ continuing professional development using their own case studies. Moreover, the platform might be used to upskill patient families and carers, which would strengthen community care and lessen the health care burden due to staff shortages [51]. Potentially, the BHS would facilitate reducing the health inequities in terms of accessing optimal health care, particularly for the economically disadvantaged or those living in remote areas.

In summary, adopting the BHS model may assist Vietnam in addressing the health crisis and achieving the country’s health care goals in the new decade. This model can increase patient accessibility to health care facilities and state-of-the-art health management, including the most vulnerable and hard-to-reach people, thus enhancing health equity across the country. It can also increase operational efficiencies for both health care providers and users, resulting in lessening overcrowded hospitals and enabling coordinated health care, which is currently missing [3,52]. Moreover, digital solutions can assist teaching, tertiary, or specialized hospitals to deliver training or conduct eHealth consultations with satellite, primary, or secondary hospitals. Therefore, the quality of health care would be strengthened across the country.

### Weaknesses

Although the BHS model has many strengths, we need to articulate inherent weaknesses in the model. First, the high cost and complexity of implementing digital health information systems, such as EMRs, may be a barrier to broad dissemination. Second, the digitalization of data and services represents a potential cybersecurity threat to privacy and trust of people in a new health care system [3]. Finally, the establishment of nationwide unique health IDs (unique codes used to identify individuals within the health care system) often takes time, especially for socially disadvantaged members of the community.

### Opportunities

Vietnam has a high-level policy framework (ie, political and legal environment) supporting the transition toward the BHS. The 2017 Resolution 20-NQ/TW of the Communist Party of Vietnam has provided a strategic orientation for reforming the health care system. This includes systemic implementation of information technology in management of primary health care, prevention, disease management, and the establishment of electronic health records (EHRs) for all citizens that link to their health insurance card.

Based on this strategic orientation, the Vietnamese Ministry of Health (MOH) in 2017 set out national goals for the protection, care, and improvement of people’s health in the period to 2030. One of the 5 key priorities for action to achieve the national health care goals was developing human resources, medical sciences, and technology [4,53]. Since then, a national agenda for digitalization of the health care system has been driven by MOH with a number of initiatives that aim to adopt digital solutions in health care network across the country. Vietnam is rapidly embracing digital health. Digitalized health care is perceived as a useful solution that could help to address the rapidly growing gap between service demand and capacity [44].

To implement health care digitalization, MOH issued Circular 54/2017/TT-BYT regarding assessment criteria for information technology applications in health care facilities [50] and a plan to develop smart health care during the period 2018-2025, with a vision toward 2030 [54]. In this plan, targeted smart health outcomes were specified, such as development of a national health care data center, electronic health and medical records, and electronic government and smart medicine management systems. Particularly in the field of examination and treatment, telemedicine is regulated by Circular 49/2017/TT-BYT, which provides guidelines for a range of telemedicine consultations in Vietnam (eg, allowing doctors to provide telemedicine services to patients under certain infrastructure requirements [55]).

To accelerate the application of information technology in the health sector, in 2018, MOH issued Circular 46/2018/TT-BYT regulating EMRs. According to Häyrinen et al [56], EMR was defined as “a repository of patient data in digital form, stored and exchanged securely, and accessible by multiple authorized users. It contains retrospective, concurrent, and prospective information, and its primary purpose is to support continuing, efficient, and quality integrated health care.” In the circular, EMRs include inpatient records, outpatient records, and other medical records prescribed by MOH, such as traditional medicine medical records and medical records of abortion. This is the first time Vietnam has specific guidance for EMR establishment, use, and management at health care facilities [57]. As the health care system in Vietnam is centralized under MOH and provincial health authorities, the integration of EMRs within the existing system, at least in the 47 central-level hospitals and possibly also at 419 provincial-level hospitals [58], will be easier than in countries with a more fragmented health care system.

The plan for implementing EHRs was approved by MOH in 2019 in Decision 5349/QĐ-BYT [59]. According to this decision, EHRs are medical documents that record the health care process of a person from birth to death in the form prescribed by MOH. This plan guides and directs all 61 cities and provinces of Vietnam to simultaneously deploy and make EHRs available for use by 80% and 95% of population in 2020 and 2025, respectively. If this aim is achieved, Vietnam will be perfectly positioned to adapt and adopt the BHS model. EHRs may be able to assist every citizen to understand and manage their health information continuously for life, enabling them to prevent disease proactively, and actively manage their health conditions if used as the basis for a BHS. EHRs will also support physicians in providing health care for people in real time and according to best practice health care recommendations. Importantly, EHRs will provide complete, accurate, and timely data on population health, enabling policy makers to advance evidence-based health policy and health authorities to examine the relative efficacy of treatments to manage health care expenditure more efficiently.

Supportive social and technological environments also place Vietnam in a good position to adopt advanced digital health solutions of the BHS. Today, Vietnam is benefiting from a golden population proportion with 70% of the population being of working age (aged 18 to 65 years) [6]. This generation is rapidly embracing new communication technologies. In 2020, Vietnam was the seventh highest number of Facebook users in the world [60]. On average, Vietnamese people spend about 7 hours per day on the internet [61]. The high uptake is associated with a high acceptability of digital technologies by Vietnamese people. Studies have shown that people’s attitudes toward mobile health solutions is highly positive. Two-thirds of Vietnamese youth and adolescents found mobile health apps useful [62,63].

Online communication is backed by a rapid and strong development of the country’s information and communication technology infrastructure: penetration rate of internet access is 67%, which is among the highest in the Asia Pacific region [64], and 95% of households are now able to use 4G network [3]. Vietnam’s technology infrastructure is also embracing cloud-based services, which will facilitate innovative and cost-effective health care delivery solutions. In recent years, a number of projects have initiated and implemented digital health services in large urban hospitals, including teleradiology, teleconsultation, telediagnosis, and videoconferencing. Examples of these initiatives include raising disease awareness and encouraging people to adopt heathier behaviors [65-69], improving accessibility to health care services among disadvantaged and vulnerable target groups [70-72], and upskilling health care workers [73-80].

A supportive economic environment has resulted in an increased need for high-quality health care services and precision medicine. Strong economic growth with an average annual gross domestic product growth rate of 6.4% [81] is creating a booming middle class in the society. It is estimated that this population will increase sharply from 10% in 2015 to more than 50% in 2035 [82]. The growth in disposable income among digitally literate, educated, and wealthy individuals creates economic conditions for higher personal expenditure on easily accessible, high-quality health care. Public health facilities often do not meet their needs due to long waiting times, inadequate consultation time, overcrowding, and high-occupancy bed rates [3]. This has resulted in the rapid expansion of a private health system, which was projected to grow from US $6.6 billion in 2016 to US $8.7 billion in 2020 [83]. Smart solutions that use big data, cloud computing, and mobile technology are strongly encouraged to alleviate overcrowded public hospitals and increase quality health care overall [4].

Together, all these supportive policy, social, technological, and economic environments provide a good foundation for the health care system in Vietnam to shift toward innovative models of care that are being proposed in developed countries, such as the BHS, which focus on patient-centered care.

### Threats

In Vietnam, patient medical records remain paper-based at all levels of the health care system and currently are required for legal purposes [84]. Moreover, although electronic administration management has been adopted in some large hospitals, the quality of medical records and databases varies across hospitals and clinics. In most health care facilities, the medical record system is not centralized [52]. As such, one patient can have multiple medical records. This is a barrier for timely medical information exchange and sharing between hospitals [69]. In addition, the development of EMRs and EHRs, a precondition for BHS adoption, is still in the early stages [4,44]. Although MOH has taken initial steps toward the development of EMR and EHR systems, the readiness to implement the EMR nationally in real clinical practice still requires considerable preparatory effort.

First, Vietnam needs to assign a unique patient identifier to every citizen, which is a core requirement for successfully introducing EHRs [52]. In 2019, MOH issued Decision 4376/QĐ-BYT, regulating the establishment, use, and management of IDs [85]. According to the decision, an ID will be created, comprising two series of numbers separated by a dot. The first series is the social insurance code of the individual while the second series is a product of an algorithm of administrative information including the social insurance code, full name, date of birth, gender, and place of birth. The social insurance, however, is a compulsory income protection insurance for employed people only to fully or partially offset their income that is reduced or lost due to sickness, maternity, labor accident, occupational disease, retirement, or death [86]. Although Vietnam has made significant progress in expanding social insurance coverage in recent years, enrollment rates are still low, especially in small- and medium-size enterprises due to multiple barriers such as cost of contribution, lack of trust in the government system, and administrative factors [87]. Despite large increases in government subsidies, low enrollment rates in compulsory social and health insurance still persist [88]. In May 2020, only 27% of Vietnam’s workforce had social insurance [89,90], which is far short of the government’s target of 50% social insurance coverage [87] and 80% health insurance coverage by 2020 [91,92]. The IDs, when established, therefore, will only cover a small proportion of the population.

Second, although many health workers and patients are aware of the advantages of using EMRs and EHRs, there are concerns about the risks of privacy violation. The Law on Network Information Security No. 86/2015/QH13, enacted in 2015, includes a requirement of “providing an adequate level of protection for personal data, following the technical standards for protection of personal data.” However, there are no clear definitions of “an adequate level of protection” and “technical standards” [93]. As such, there is a lack of regulatory rigor and sanctions in managing data processors. Strengthening the legal and regulatory system to protect patient privacy and information security is fundamental for the success of EMR development and application.

Finally, since Vietnam is still in the very early stage of health care digitalization, there is no empirical evidence about the effectiveness and sustainability of digital health initiatives. Over the past few years, there have been a growing number of organizations and health startups delivering digital health solutions to improve the quality of medical services. For example, eHospital software, developed by the Corporation for Financing and Promoting Technology, was first launched in 2000 [94]. This is a comprehensive hospital management system providing supports to manage all activities relating to patients in health care facilities. In 2018, eHospital with the application of the latest 4.0 technologies such as artificial intelligence and big data was introduced. Until now, this system has been used in more than 400 hospitals and clinics in Vietnam. [95].

Another example is the Hospital Information System of the Vietnam Posts and Telecommunications Group, which was introduced in 2015. This solution with its three levels of management—state, hospital and patient—aims at supporting provincial health authorities and hospital managers to make well-informed decisions for health care improvement and assisting patients to facilitate and adhere to their health care appointments online. This allegedly leads to increasing transparency and reducing overcrowding in health care facilities [96]. Nevertheless, these initiatives have not been rigorously evaluated. Lack of evaluation results will prevent the government from evidence-based policymaking and hinder the broader implementation of existing projects or development of new initiatives.

## Discussion

### Necessary Conditions for Successful Adoption of the BHS Model in Vietnam

Vietnam has identifiable opportunities to adopt the BHS model and implement digitalization in health care. To grasp these opportunities, the following strategies are recommended.

First, establishing a national ID for each individual based on their social insurance code is one core requirement for successfully introducing EHRs. In a recent Organisation for Economic Co-operation and Development policy report, recommendations have been proposed for the Vietnamese government to increase the enrollment rates of social insurance. They include reducing obstacles to participation (eg, simplifying administration processes for paying insurance), introducing incentive scheme for employees to participate (eg, providing government subsidies for participation of low-income people in the voluntary system), and adopting a systematic approach to social protection (eg, considering the interaction between various social protection mechanisms) [87].

Second, successful adoption of the BHS requires a whole-system approach involving the support of different sectors in the society. Obviously, MOH would have to play a key role in this adoption process but would need to collaborate with related ministries—in particular, the Ministry of Science and Technology and the Ministry of Information and Communications—in developing and implementing a digital health strategy. The resources needed to improve the health care system are sizeable. MOH would need to garner funding from different sources, both domestic and foreign agencies: government, social entrepreneurs, or private businesses [69,70].

Third, integrating personal data in EHR and EMR systems will increase the risk of privacy violation and cybersecurity breaches. The Vietnamese government needs to improve the security of information technology platforms in general and health care in particular to protect patient privacy and information security [70].

Fourth, to improve the adoption of evidence-based practices, it will be necessary to provide resources to demonstrate the effectiveness and impact of digital health initiatives and establish a network of collaborators including health care administrators, clinicians, community representatives, digital health researchers, information technology developers, and public health education experts [69]. This will be a great opportunity for further enhancing strong collaborations between multisectors and multistakeholders at various levels, which are essential for successfully reforming the health care system in Vietnam. BHS implementation would require the involvement of local educators to provide education for communities, raising awareness of the benefits the system could bring to individuals and society, coaching to enhance self-management behaviors, and increasing engagement with the system [97]. Strong evidence about the effectiveness of digital health initiatives in Vietnam will encourage the government to develop appropriate health policies and increase opportunities for ongoing projects to scale-up by attracting funding and support [70]. Thus, there is a substantial need for further research in this area in the future.

Finally, to increase the acceptability and feasibility of digital health initiatives, a co-design approach is crucial. Co-design is defined as the “collective creativity as it is applied across the whole span of a design process” [98]. In the design process, diverse experts such as researchers, designers or developers and potential customers and users work closely together to understand the needs and preferences of end users [99,100]. A co-design approach is likely to increase scalability and dissemination of the initiative [99].

## Conclusions

The overarching goal of the BHS is, with the support of digital technologies, to deliver best practice health care and reduce pressure on the current health care system by empowering people to direct their own health care, regardless of their geographical location and economic status [44]. The BHS offers a promising and intelligent health care that which may be efficient and suitable for Vietnam in the new era of the Fourth Industrial Revolution [101]. Vietnam has tremendous opportunities and a favorable policy, social, technological, and economic environment to adopt this model of comprehensive patient-centric health care. In order to uptake, adapt, and implement the BHS successfully, Vietnam needs to apply a whole-system approach in transformation and operation processes.

## Abbreviations

BHS: Bespoke Health Care System
EHR: electronic health record
EMR: electronic medical record
HIS: health information system
MOH: Ministry of Health of Vietnam
NCD: noncommunicable disease
OPP: out-of-pocket payment
SWOT: strength, weakness, opportunity, and threat analysis
THE: total health expenditure
WHO: World Health Organization
